# Echocardiographic assessment of ischemic mitral regurgitation

**DOI:** 10.1186/1476-7120-12-46

**Published:** 2014-11-21

**Authors:** David M Dudzinski, Judy Hung

**Affiliations:** Echocardiography Laboratory, Cardiology Division and Division of Critical Care Medicine, Massachusetts General Hospital, Boston, MA 02114 USA; Echocardiography Laboratory, Cardiology Division, Massachusetts General Hospital, Boston, MA 02114 USA

**Keywords:** Chronic Ischemic mitral regurgitation, Echocardiography, Ventricular remodelling, Papillary muscle displacement, Mitral valve tethering, Mitral valve tenting, Tethering angles, Tenting height, Tenting area, Mitral annulus dilatation, Myocardial infarction

## Abstract

Ischemic mitral regurgitation is an important consequence of LV remodeling after myocardial infarction. Echocardiographic diagnosis and assessment of ischemic mitral regurgitation are critical to gauge its adverse effects on prognosis and to attempt to tailor rational treatment strategy. There is no single approach to the echocardiographic assessment of ischemic mitral regurgitation: standard echocardiographic measures of mitral regurgitation severity and of LV dysfunction are complemented by assessments of displacement of the papillary muscles and quantitative indices of mitral valve deformation. Development of novel approaches to understand mitral valve geometry by echocardiography may improve understanding of the mechanism, clinical trajectory, and reparability of ischemic mitral regurgitation.

**Electronic supplementary material:**

The online version of this article (doi:10.1186/1476-7120-12-46) contains supplementary material, which is available to authorized users.

## Background

The mitral valve has a specific geometry designed to maintain leaflet coaptation and thereby prevent systolic regurgitation into the left atrium (LA). Mitral valve function must be conceptualized in terms of a holistic relationship with supporting ventricular structures, and thus derangements of any part of the mitral valve apparatus – including the mitral valve leaflets, but also the annulus, chordae tendinae, papillary muscles (PM), and left ventricle (LV) – can disrupt valvular coaptation and cause symptoms, physical examination findings, and echocardiographic manifestations of valvular incompetence. The concept of ischemic mitral regurgitation must be clarified in terms of possible mitral regurgitation (MR) mechanisms as well as by acuity of the insult causing MR (see Table 1). In acute coronary syndromes and early in the course of myocardial infarction, MR may occur due to PM ischemia or rupture due to infarction, ischemic LV dilation, and/or increased LV diastolic pressures.

**Table 1:** **Classification of mitral regurgitation by mechanism and acuity**

	Primary (“Organic”)	Secondary (“Functional”)
**Acute**	Papillary muscle ischemia	Acute ischemic LV dilatation
	Ruptured papillary muscle (trauma, infarction)	
	Flail mitral valve leaflet	
	Ruptured chordae tendinae	
	Endocarditis (leaflet perforation)	
**Chronic**	Flail mitral valve leaflet	**Chronic ischemic mitral regurgitation (CIMR)**
	Mitral valve prolapse	Non-ischemic LV dilatation (failure of leaflets to coapt)
	Ruptured chordae tendinae	Non-ischemic LV systolic dysfunction
	Degeneration (myxomatous, endocarditis, calcification)	Hypertrophic cardiomyopathy
	Rheumatic	Right ventricular pacing
	Congenital	Aortic insufficiency [1]

The most common clinical situation encountered for MR arising post infarct, e.g. chronic ischemic mitral regurgitation (CIMR, also called “ischemic chronic secondary MR" by new guidelines [2]), is MR due to geometric changes of the LV and distortion of normal spatial relationships of the mitral apparatus, all secondary to remodeling from ischemic heart disease. CIMR is characterized mechanistically by incomplete mitral leaflet closure, namely displacement of the leaflet coaptation apically within the LV cavity [3]. Although a spectrum of anatomic abnormalities of both LV and PMs exists, evidence points to a predominant role of “tethering” as the final common pathway in inducing CIMR. Post-infarct, outward displacement of PMs leads to stretching of the chordae tendinae and increased tethering forces on the mitral leaflets, which causes the apical coaptation and restricted closure. Annular dilatation may also contribute by stretching leaflets and causing incomplete closure. Accordingly, CIMR is classified as functional MR, or type IIIb in the Carpentier classification. Practitioners should also be mindful not to classify as CIMR those patients with mitral regurgitation and comorbid ischemic heart disease if there is any intrinsic mitral valve apparatus abnormality and/or there has not been a history of myocardial infarction.

This article will focus specifically on the echocardiographic characteristics of CIMR – given its importance in adverse prognosis (e.g. heart failure and mortality [4, 5]) and impact on evaluating treatment decisions including revascularization, annuloplasty, and cardiac resynchronization therapy. Echocardiography is the only reliable method available for clinical evaluation of CIMR because the physical examination reveals no audible murmurs in about one-third of CIMR patients with moderate or severe MR and half with mild MR [4].

### Overview of echocardiographic assessment of CIMR

When assessing MR by echocardiography, the key inquiries are severity, delineation of pathology in the components of the mitral valve apparatus, overall mechanism of MR, and, based on the probable mechanism, consideration of treatment strategies to reduce MR. As applied to CIMR, the echocardiographer should:

1. confirm underlying chronic ischemic heart disease,
2. gauge the severity of MR,
3. exclude intrinsic pathology in the leaflets and chordae,
4. establish CIMR as the most likely etiology by assessing LV and PM displacement, and
5. characterize the phenotype of CIMR as either symmetric or asymmetric.

Echocardiographic assessment of CIMR should also include assessment of global and regional LV function, LV ejection fraction, LV dimensions, LV wall motion abnormalities, and pulmonary hypertension [2].

The prevalence of post-infarct MR has been reported to be as high as 50% in patient populations studied by echocardiography within 7 to 30 days post infarct [4, 5]. Thus echocardiography may be used to confirm sequelae of ischemic heart disease such myocardial scarring, wall thinning, and wall motion abnormalities. However, it is incumbent on the cardiologist and echocardiographer to be familiar with the patient’s history and other available diagnostic results including electrocardiography, nuclear perfusion tests, and angiography.

**Figure 1:**
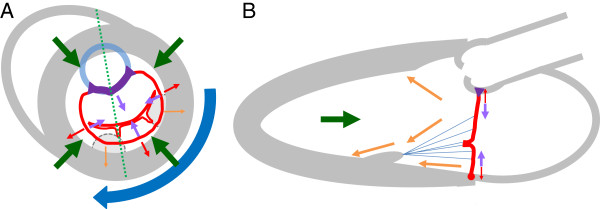
**Model of closing and tethering forces acting on the mitral valve.** The forces acting on mitral valve leaflets are shown in model parasternal short axis **(A)** and long axis **(B)** sections (green dashed line in **(A)** indicates the plane shown as **(B)**). The LV, LA, and aorta are shown in gray and blue, with PMs indicated by hatched lines (since the PMs are not at the same level as the mitral annulus). The mitral annulus and leaflets are shown in red with the aortomitral curtain in purple. Normal systolic closing forces include: LV contraction (green arrows), basal myocardial clockwise rotation (blue arrow), and mitral annulus contraction (purple arrows). Tethering forces include passive constraint of the mitral annulus (red arrows) and tethering from the PM-chordae and PM contraction (orange arrows). The relative size of the arrow delineates relative magnitude of the forces acting on the mitral leaflets.

Concomitant with measuring severity of MR, one of the initial jobs of the echocardiographer is to ensure there is no other intrinsic pathology of the leaflets, chordae, and PMs; identifying such a finding could indicate the mechanism may not be CIMR. In general the pathologic processes underlying CIMR reflect ventricular and not leaflet pathology: adverse local and global remodelling of the LV changes the geometry of the PMs and the resultant dynamic vector forces exerted on the chordae-leaflet system. During systole, mitral leaflet closing is mediated by the interplay of closing forces [6] exerted by LV intracavitary systolic pressure on the ventricular surface of the mitral leaflets versus tethering forces, which restrict leaflet motion in systole by pulling apically away from the mitral annular coaptation plane. Tethering forces are applied by the LV, PMs, and annulus along apical, posterior, and lateral vectors [7] (Figure 1) and cause incomplete systolic mitral leaflet closure [3]. Global LV dilatation will increase the distance from PM to the leaflet and cause tethering; similarly, a local area of infarction that distorts and outwardly displaces the myocardium underlying PM produces similar malposition. In part because of the more common single vessel supply of the posteromedial PM [8], tethering and resultant CIMR are more common with inferior infarcts than with anterior infarct [9]. In addition, displacement of the anterolateral PM is more restricted due to the structural buttress afforded by the interventricular septum, and also because infarcts in the left coronary artery territory will more commonly produce apical dilatation as opposed to dilatation of territory subtended by the anterolateral PM.

**Figure 2:**
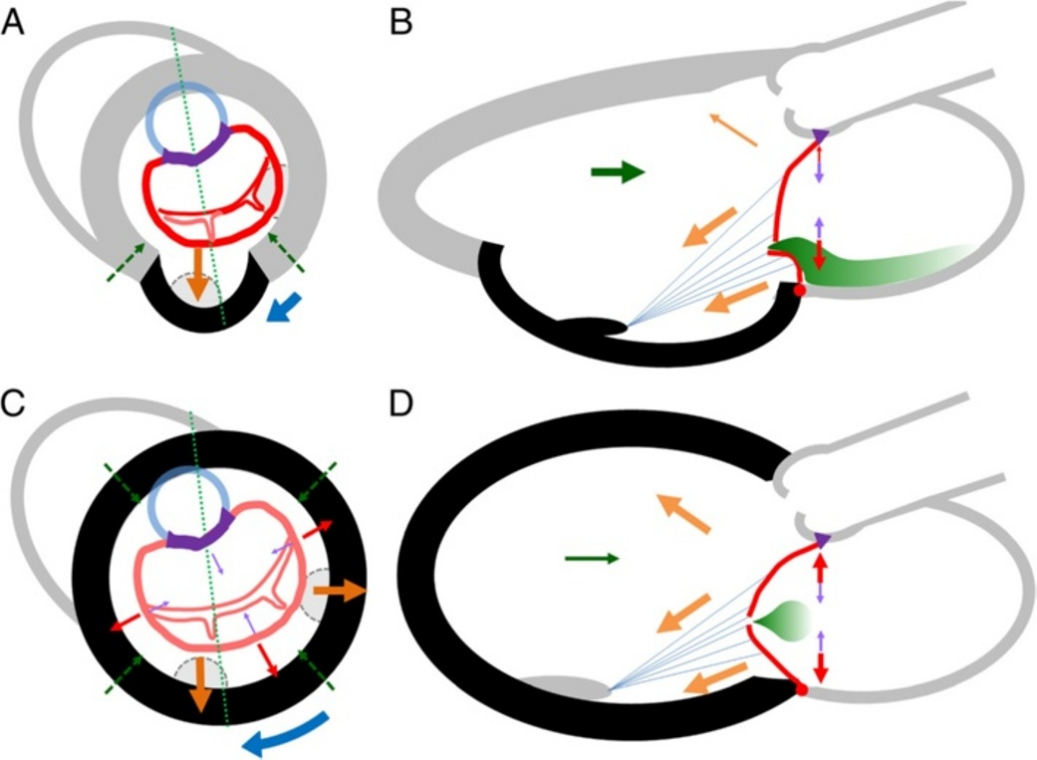
**Models of symmetric and asymmetric phenotypes of CIMR.** Figure 2 depicts two types of CIMR phenotypes, asymmetric (panels **A** and **B**) and symmetric (panels **C** and **D**), based on the model established in Figure 1, with arrows indicating the forces which have changed in magnitude. An inferoposterior myocardial infarction (black area in panels **A** and **B**) causes local outpouching of the LV myocardium in a posterior direction, which displaces the posteromedial PM and increases tethering forces exerted on the leaflets. In addition, there is less LV closing force (green arrows) and decreased basal clockwise rotation force (blue arrow). Due to posterior > anterior leaflet tethering and pseudoprolapse, there is posteriorly directed eccentric MR (green shaded area). Global LV dilatation and spherical remodelling (indicated by black areas of panels **C** and **D**) displaces both PMs with posterior, lateral, and apical vectors exerted on the mitral leaflets (orange arrows). Aggregate LV closing force is reduced (green arrow). The enlarged mitral annulus contributes to higher passive tethering force on the leaflets (red arrows) and less mitral annular contraction (red arrows). The net result is apical displacement of the mitral leaflets and their coaptation zone, with central MR.

**Figure 3:**
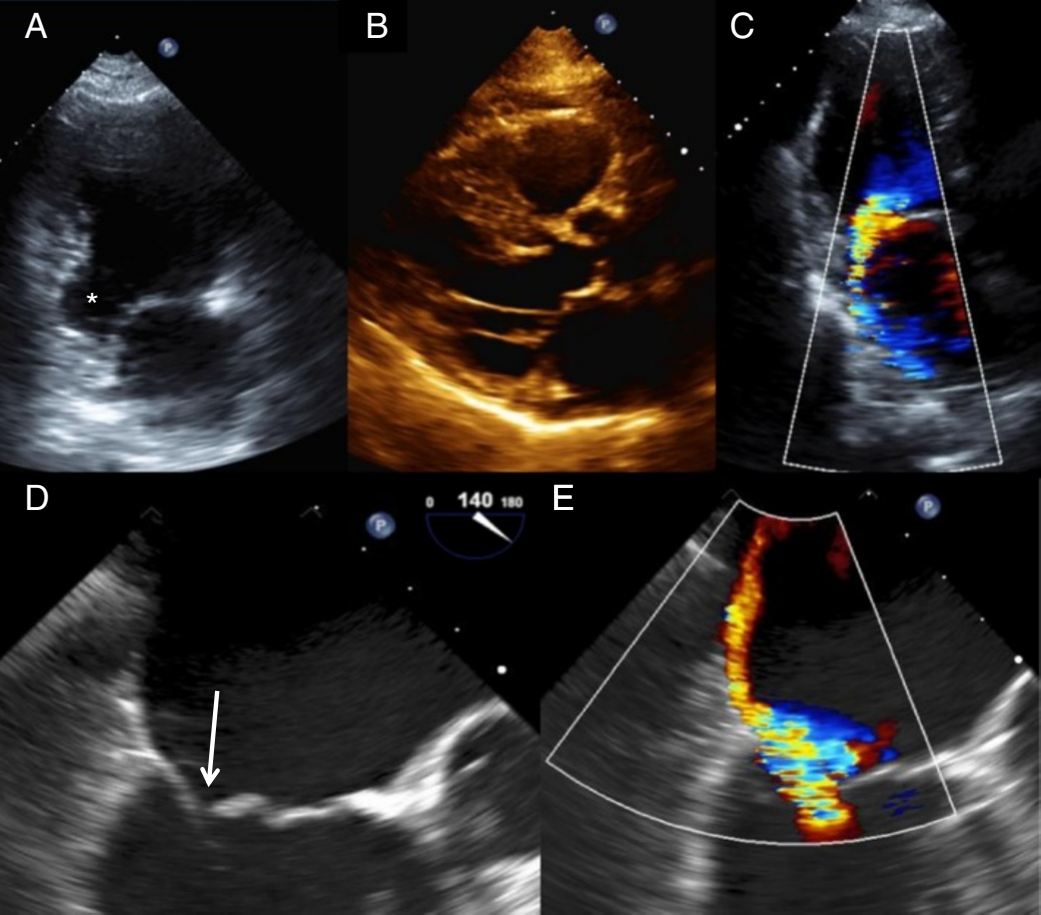
**Echocardiographic images of asymmetric CIMR due to inferoposterior myocardial infarct and posterior leaflet tethering.** These echocardiographic images were obtained from an 81 year-old male with a history of inferior and inferoposterior myocardial infarction with localized aneurysmal ventricular deformity and atrial fibrillation, when he was evaluated for dyspnea and congestive heart failure. The previous echocardiogram obtained three years prior had demonstrated mild posteriorly directed MR. The apical two chamber view at end-diastole from the current transthoracic echocardiogram shows the true inferobasal aneurysm indicated by an asterisk **(A)**. Asymmetric mitral valve leaflet tenting is depicted in the parasternal long-axis view at mid-systole **(B)**. Additional quantitative measures of tethering phenotype are described in the subsequent main text. The tenting height measured from the mitral annulus plane was 1.4 cm and the tenting area bounded by the mitral annular plane and leaflets was 4.0 cm^2^; tethering angles β and α measured approximately 55° and 40° respectively. The jet of MR was posteriorly directed and reported moderate in severity **(C)**. TEE was then undertaken to confirm the mechanism of MR and this also revealed incomplete mitral valve closure due to PM displacement (**D**: mid-esophageal long-axis view at omniplane angle 140°, image taken at mid-systole) with pseudoprolapse (arrow) of the anterior leaflet tip relative to the more adversely tethered posterior leaflet. This locus of malcoaptation is the area from which the MR originates. There is severe MR with an eccentrically directed posterior jet (**E**: mid-esophageal long-axis view at omniplane angle 140°, image taken at mid-systole) with evidence of systolic flow reversal in pulmonary veins (not shown).

The vector sum of forces applied to the mitral leaflet in CIMR generates an abnormal, ventricularly displaced coaptation shape of the mitral leaflets referred to as “tenting”. Two echocardiographic phenotypes of tenting in CIMR have been identified [7]: asymmetric and symmetric (Figure 2), which depends on if the posterior or both leaflets are affected, which itself depends on the underlying LV and PM derangements. For example, global LV dilatation with radially outward and apical displacement of both PMs causes symmetric tenting of both leaflets. Inferoposterior infarction [10] with local adverse remodelling predominantly affects the posteromedial PM and restricts the posterior leaflet motion, causing a relative overriding of the coaptation zone by the non-tethered leaflet (“pseudoprolapse”). The coaptation zone, although more apically displaced in symmetric tethering, is less geometrically deformed than in asymmetric tethering. Regurgitant severity of CIMR is strongly affected by tethering phenotype, with higher degrees and more eccentric jets observed in asymmetric tethering (Figure 3). Symmetric tethering, despite more common association with worse LV dysfunction and dilatation, more frequently results in smaller non-eccentric central jets of MR. The characteristics of these phenotypes are summarized in Table 2.

**Table 2:** **Characteristics of symmetric versus asymmetric CIMR tethering phenotypes**

	Symmetric CIMR	Asymmetric CIMR
Major net tethering vectors	Apical	Posterior > apical
Tethered mitral leaflets	Anterior and posterior	Posterior
Associated myocardial infarct pattern (and vessels)	Anterior (left anterior descending/multiple coronary arteries)	Inferoposterior (right coronary > circumflex)
Archetype pattern of LV dysfunction	Global LV dilatation and wall motion abnormality	Inferoposterior wall motion abnormality and dilatation
Mitral valve coaptation zone displacement	Apically	Posteriorly; pseudoprolapse of anterior leaflet over posterior leaflet
Tethering angles	Anterior ≈ posterior	Posterior > anterior
Tenting volume	Higher	Lower
MR origin and direction	Central, non-eccentric	Posterior, posteriorly eccentric
Annular dilatation	Greater	Lesser
Annular height	Greater loss of non-planarity (“flattened”)	Less loss of height
MR relative severity	Less severe	More severe
Severity correlates best with	LV dilatation and sphericity	Degree of mitral valve deformation (greater tethering angles, more marked derangement of coaptation zone)

In the following sections, transthoracic echocardiographic techniques will be discussed in tandem with reference to the underlying plausible mechanisms of CIMR and other etiologic contributors such as mitral annular dilatation.

#### Echocardiographic assessment of ischemic MR severity

Accurate grading of MR is central to clinical decision-making. MR should be graded using an integrative approach, incorporating multiple Doppler techniques for direct quantification as well as supportive data (left atrial size, LV chamber size, pattern of pulmonary vein flow) in the overall assessment [11]. Color Doppler techniques include:

**Figure 4:**
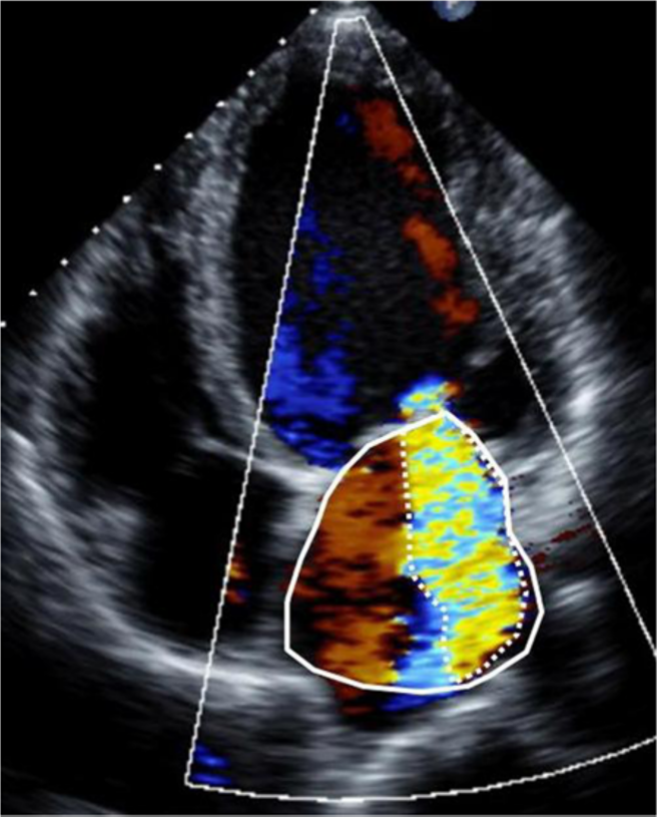
**MR quantification by ratio of maximal distal jet area to left atrial area.** Assessment of MR severity by distal jet area involves tracing the jet area (dashed white line) in the apical four chamber view and comparing the ratio of jet area to the left atrial area (solid white line). See Tables 3 and 4 for MR severity grades corresponding to different jet area:left atrial area ratios.

**Figure 5:**
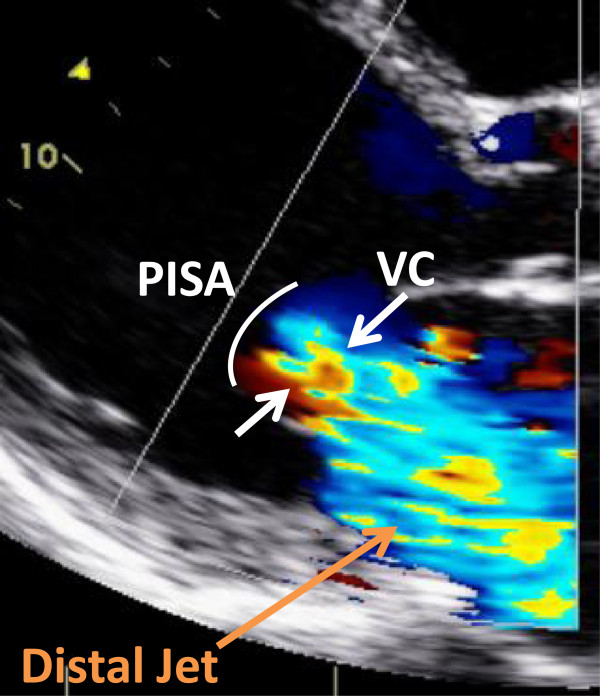
**Vena contracta measurement.** The VC (white arrows) of the MR jet is measured from parasternal long axis view as the narrowest width of the proximal jet at the level of or just distal to the leaflet tips. In this view, the image is zoomed into the area of the VC (arrows), with sector size and depth selected to optimize color Doppler resolution. Magnification is essential to correct grading of MR severity by the VC method because small changes in the measurement may affect severity grade. VC = vena contracta.

A. Distal jet area measures the high turbulent mosaic color Doppler pattern produced by the MR flow as it enters the left atrium, distal to the mitral valve leaflets. This color Doppler display is a surrogate measure of MR volume. It is measured as an absolute area, or also as a ratio relative to the left atrial area. The MR jet area is traced at its maximum in apical views and divided by the left atrial area traced in same frame (Figure 4). The advantage of jet area ratio is that it is a rapid, straightforward method, especially for centrally directed MR jets. Its disadvantages are that the distal MR jet varies with loading conditions, such as blood pressure, or technical factors such as machine color gain and frequency settings. In addition, the MR volume in eccentric jets is underestimated by the distal jet area method as the jet can be attenuated by the left atrial wall.B. Vena contracta (VC) measures the linear dimension of the neck of the MR jet as it enters the regurgitant orifice at the level of the leaflets. The VC is a simple linear measure of the regurgitant orifice and is relatively independent of loading conditions. The VC is measured in the parasternal long axis plane with the VC region magnified, and depth and sector size optimized for color Doppler resolution (Figure 5). Magnification is critical for accurate vena contracta grading, as small differences in measurement may change in grading category. Because reference ranges for VC have been defined in long axis planes, measurement of VC in the 2 chamber view should be avoided.

**Figure 6:**
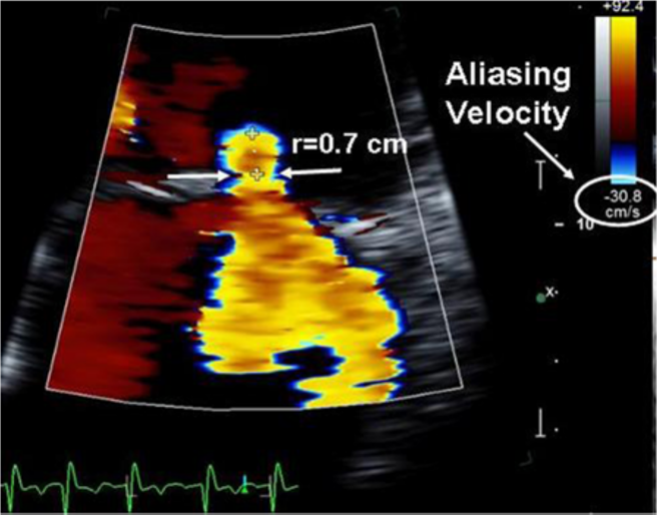
**Calculation of EROA by PISA method.** In this apical four chamber view, the PISA region is displayed from a magnified apical view, and the hemispheric PISA radius R is shown between the crosshairs. Note the change of the color Doppler scale, with a baseline shift in the direction of the MR jet (e.g. “down” in this example). The aliasing velocity is 30.8 cm/s. EROA can be calculated as the product of 2πR^2^ x Aliasing Velocity ÷ Peak Velocity of MR (peak velocity not shown). The vena contracta is indicated as the distance between the two arrows. EROA = effective regurgitant orifice area; PISA = proximal isovelocity surface area.

C. Proximal isovelocity surface area (PISA) or proximal flow convergence method calculates the effective regurgitant orifice area (EROA) and MR regurgitant volume (RVol) as follows: 

where R is the radius of the hemispheric PISA zone (Figure 6)


The PISA method provides a quantitative method for MR grading. However, the calculation requires a geometric assumption of a hemispherical shape to the PISA region which is not always the case. Additionally, it can be technically challenging to measure the PISA radius accurately.

D. The pulsed Doppler volumetric method calculates MR RVol as mitral valve inflow minus aortic outflow. Although it provides a quantitative measure of MR, it has multiple measurement steps, each with potential for measurement variability and error. Additionally, this method requires two non-stenotic valves without important aortic insufficiency.

Tables 3 and 4 shows reference ranges for color Doppler criteria for MR grade based on 2003 American Society of Echocardiography guidelines [11]; however 2014 American College of Cardiology/American Heart Association guidelines propose a new classification scheme of valvular disease severity, based on a combination of echocardiographic and symptomatic parameters, with stages of “at risk” to “progressive” to “asymptomatic severe” to “symptomatic severe” [2]. Recent consensus statements also endorse lower cut-off values for EROA for CIMR severity as compared to primary MR. In part this is due to 1) data that shows worse prognosis at smaller EROA in CIMR, likely reflecting the effects of the incremental volume load of lesser degrees of MR on an already dysfunctional ventricle, and 2) 2D echocardiographic underestimation of the flow convergence-method derived EROA due to “crescentic” orifice geometry in CIMR as opposed to a circular orifice [2].

**Table 3:** **Guideline based reference ranges for grading mr 2003 ASE guidelines**

2003 ase guidelines			
Parameter	Mild	Moderate	Severe
*EROA-CIMR (cm* ^*2*^ *)*			≥ 0.2
*EROA-Primary MR (cm* ^*2*^ *)*	< 0.2	0.2-0.39	≥ 0.4
*VC width (cm)*	< 0.3	0.3-0.69	≥ 0.7
*Jet/LA area*	< 20%	20-39%	≥ 40%
*MR Reg. Volume*	< 30 ml	30-59 ml	≥ 60 ml

**Table 4:** **Guideline based reference ranges for grading mr 2014 AHA/ACC Guidelines**

2014 AHA/ACC Guidelines				
Parameter	Stage A “At risk”	Stage B “Progressive”	Stage C “Asymptomatic Severe”	Stage D “Symptomatic Severe”
*Valve apparatus and anatomy*	CAD or cardiomyopathy, with normal valve leaflets, chords, annulus	Regional WMA with mild tethering of MV	Regional WMA ± LV dilatation with severe tethering of MV	Regional WMA ± LV dilatation with severe tethering of MV
		Annular dilatation with mild loss of central coaptation	Annular dilatation with severe loss of central coaptation	Annular dilatation with severe loss of central coaptation
*LV (ischemic or primary myocardial disease)*	No or mild dilatation with infarct or inducible ischemia, or cardiomyopathy with LV systolic dysfunction and dilatation	Regional WMA with reduced LV systolic function ± dilatation	Regional WMA with reduced LV systolic function ± dilatation	Regional WMA with reduced LV systolic function ± dilatation
*Symptoms*	May be present, may respond to GDMT	May be present, may respond to GDMT	May be present, may respond to GDMT	Symptoms persist despite GDMT
*EROA-CIMR (cm* ^*2*^ *)*	< 0.2	< 0.2	≥ 0.2	≥ 0.2
*Jet/LA area*	No MR jet or jet area/LA area <20%	20-39%	≥ 40%	≥ 40%
*VC width (cm)*	< 0.3		≥ 0.7	≥ 0.7
*Regurgitant Fraction*		< 50%	≥ 50%	≥ 50%
*Regurgitant Volume*		< 30 mL	≥ 30 mL	≥ 30 mL

**Figure 7:**
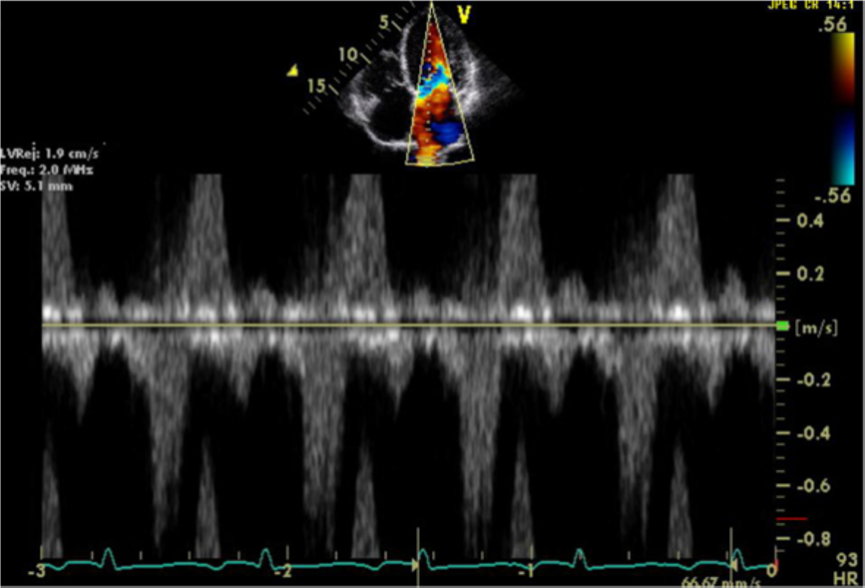
**Reversal of pulmonary vein flow.** Pulsed wave Doppler interrogation of the right upper pulmonary vein in this apical four chamber view shows systolic reversal of flow. This is a specific, albeit lower sensitivity, sign of severe MR.

**Figure 8:**
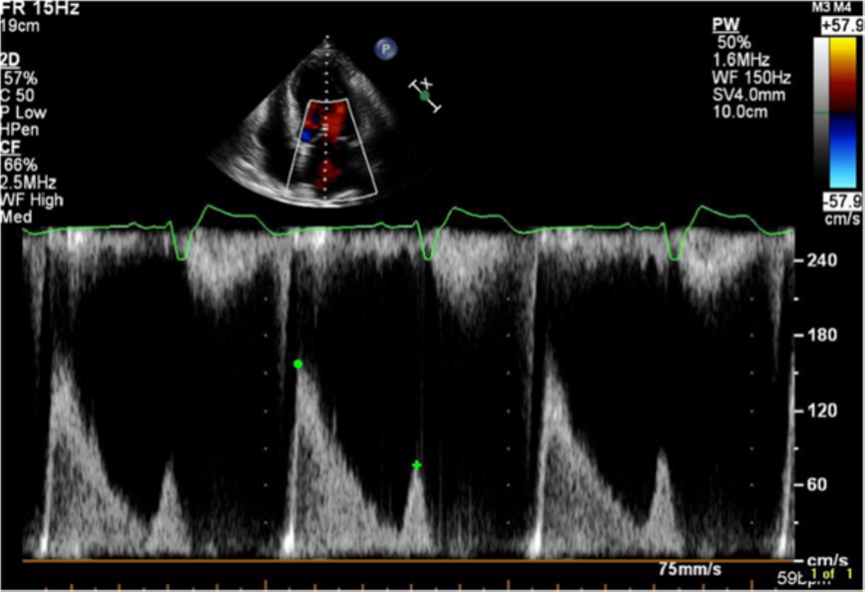
**Pulsed wave Doppler of transmitral flow.** Pulsed wave Doppler interrogation from the apical four chamber view of the transmitral diastolic flows into the LV can provide adjunct information to grading of MR severity. In this example, the E wave measures approximately 1.6 m/s, and this is consistent with a high flow rate of early diastolic passive LV filling which can be seen with severe MR.

**Figure 9:**
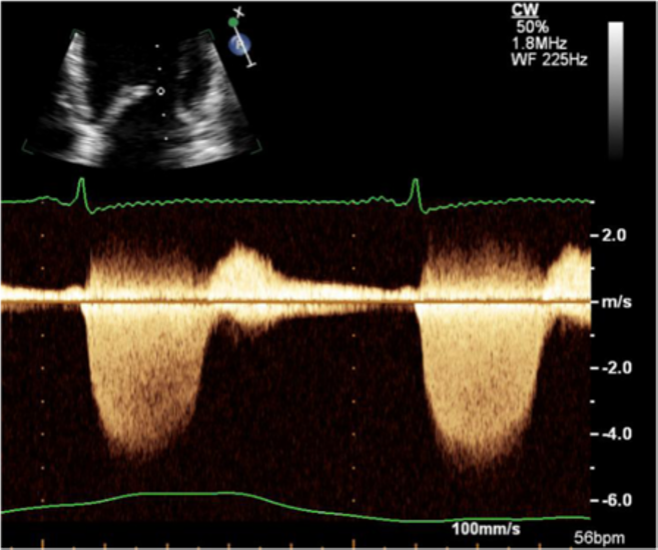
**Dense Doppler Signature in Severe MR.** This continuous wave Doppler interrogation of an MR jet taken from the apical four chamber view shows a very dense Doppler profile, which is consistent with severe MR. The peak MR velocity is 4.5 m/s, and this value would be used in the denominator of the calculation of EROA.

In addition to semi-quantitative and quantitative Doppler techniques, it is important to integrate supportive and complementary data into the overall severity grading. Pulmonary venous flow reversal is specific for severe MR although of lower sensitivity (Figure 7). Chamber enlargement (LA and LV), dense continuous wave MR Doppler profile, and elevated E wave peak velocity >1.2 m/s are all suggestive of severe MR [11–13] (Figures 8 and 9).

3D echocardiography has been demonstrated to provide accurate and reproducible MR grading using 3D guided planimetry of the VC area, which is essentially equivalent to the direct measure of the EROA. An advantages of 3D measurement of the EROA is that it does not require geometric assumptions that are used for 2D EROA calculation. Disadvantages are the lower frame rates of 3D color Doppler, which can effect lateral resolution and hence may erroneously exaggerate the area measured [14].

Finally, CIMR is a dynamic process, and the echocardiographer must consider how ambient preloading and afterloading conditions such as patient’s volume status, systemic blood pressure, and medications may affect the observed degree of MR.

#### Echocardiographic assessment of global LV enlargement and dysfunction

Quantitative measures have attempted to correlate LV systolic dysfunction and LV dilatation with CIMR. Elegant experimental observations show that isolated LV systolic dysfunction (pharmacologically induced in a large animal model) does not produce significant MR [6, 15]. This is likely because without tethering forces, relatively little closing force is required to be generated by the LV to force the mitral leaflets toward the annular coaptation zone. However, in the same model of pharmacologic LV systolic dysfunction, when the LV was allowed to dilate by relieving an extrinsic pericardial restraint, MR was generated. This observation confirms as a key mechanism the apical and outward dilatation of PMs which create tethering forces on the leaflets.

In CIMR with symmetric tethering, LV end-systolic and end-diastolic volumes and the sphericity index correlate with the severity of MR. This is because the degree of LV dilatation directly relates to apical displacement of the PMs. For asymmetric tethering phenotypes the measures of global LV remodeling do not as robustly correlate with severity of MR because a small infarct can disrupt PM geometry and generate severe MR; the actual measures of mitral valve deformation are better predictors (see below). LV dilatation would therefore not be an independent predictor of CIMR severity in a population with mixed CIMR phenotypes.

#### Local LV remodelling and PM displacement

The normal orientation of the PMs is with their long axis parallel to that of the LV and perpendicular to the plane of the mitral annulus. A local infarct that disrupts myocardium underlying a PM can radically change the relationship of that PM relative to the other PM and to the valve apparatus. This asymmetric effect of the infarct on the posteromedial PM translates directly into creating asymmetry in mitral valve apparatus anatomy and function – by rotating the posteromedial PM, tethering the posterior leaflet, and deforming the posterior portion of the mitral annulus – which creates a substrate for eccentric CIMR (Figure 3 and Additional file 1). Several lines of experimental and echocardiographic evidence correlate post-infarct inferoposterior wall motion abnormality with severity of MR [16]. Direct evidence that PM displacement generates CIMR was obtained in a sheep study of echocardiography-guided PM repositioning by an inflatable balloon external to the myocardium [17]. In this study, a Dacron patch with an adjustable balloon was sewn epicardially over areas of infarct after circumflex artery ligation; inflation of the balloon could be tailored to reduce the ischemic dilatation of the inferior wall, thus re-approximating PM geometry, and reducing severity of MR without a change in measures of LV contractility.

**Figure 10:**
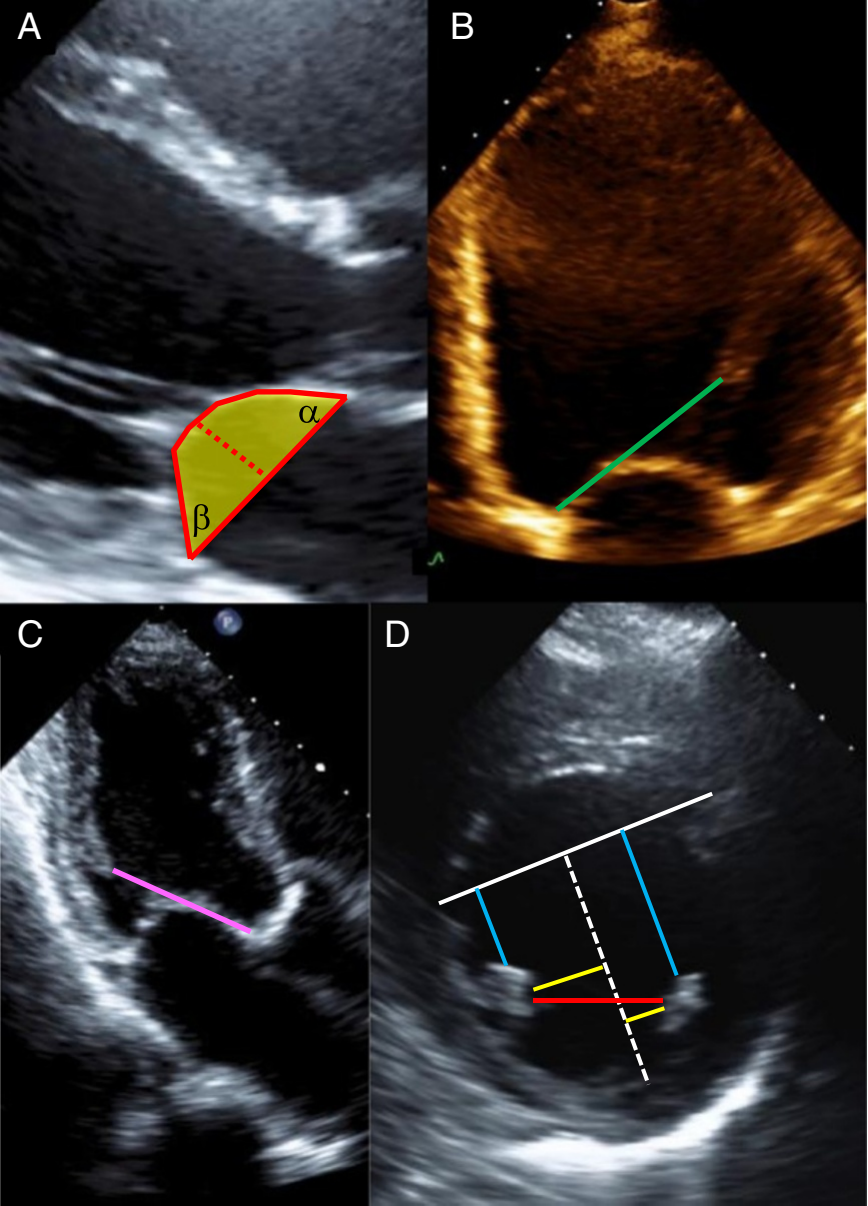
**Representative measurements of tenting height, tenting angles, and tenting area.** Panel **A** shows the measurement of tenting height, tenting area, and tenting angles in a mid-systolic parasternal long axis TTE image. The mitral annulus line is drawn and the distance from the annulus line to the coaptation point (red hatched line) represents tenting height. The posterior and anterior leaflets’ silhouettes are traced from the annular line to the coaptation zone to delineate an area (yellow shading) that represents the tenting area. Tethering angles are measured as the angle that the mitral leaflets create with the mitral annulus line. Panel **B** shows measurement of apical displacement the anterolateral PM in the apical four chamber view (green line), and Panel **C** shows measurement of apical displacement the posteromedial PM in the apical three chamber view (pink line). Panel **D** shows a representative parasternal short-axis mid-ventricular level view in early systole with PM bodies in cross-section. A reference grid of a mid-septal perpendicular chord (white line) spanning the septal insertions of right ventricle myocardium and another line orthogonal to and originating from the center of the first line (hatched white line) was created. The mid-septal perpendicular chord allows posterior displacements of both PMs to be measured (blue lines), as well as to measure lateral displacements of both PMs (yellow lines). Intra-PM distance is shown by the red line. These distances are all elevated in CIMR compared to normal controls. Though there is significant overlap in the distances among symmetric and asymmetric CIMR phenotypes, relative displacements of the posteromedial PM are increased in asymmetric CIMR as compared to the anterolateral PM (see Table 5).

In practice, echocardiographic measurement of PM displacement requires intracardiac landmarks. The anterior mitral annulus is anchored at the aortomitral fibrous curtain, and this point in the parasternal long axis or apical four chamber views can provide a reference for measurement of apical displacement of both PM heads (Figure 10B,C) [18]. In a population of 128 LV systolic dysfunction patients, the strongest multivariate correlations with MR severity in a functional MR model were the apical displacement of the posteromedial PM and the inferoposterior displacement of the anterolateral PM [18]. In the parasternal short axis view at the mid-ventricular level, PM body displacements may be referenced relative to the mathematical center of the LV. Agricola and colleagues constructed a “mid-septal perpendicular line,” bounded by the septal insertions of the right ventricle myocardium, from which to measure posterior displacements of the PMs (Figure 10D) [7]. Lateral displacements of both PMs were measured from a second line constructed orthogonal to the mid-septal perpendicular line. Finally, a distance between the papillary body muscles was recorded. Regardless of CIMR phenotype, absolute value of each of these displacement measures is higher when compared to normal controls. In addition, the displacement measures will tend to be higher in symmetric versus asymmetric CIMR, but the magnitude of the changes between phenotypes is a few millimeters and thus not sufficient to differentiate them without other information on mitral valve deformation (Table 5). Some differences correlate with asymmetric phenotypes, e.g. the ratio of posterior displacements of the posteromedial:anterolateral PMs is about 1.2 in asymmetric CIMR but about 0.94 in symmetric CIMR or normal controls [7]. 3D TTE permits additional insight into the geometric angles relating both PMs to the LV cavity long axis, with greater asymmetry in the angles in CIMR versus functional MR with a dilated cardiomyopathy [19]. 3D TTE can be used to measure true spatial vector distances from the aortomitral curtain to the PM tips [20] and also characterize the spatial geometry of the PMs in relation to the annulus [21].

**Table 5:** **Ranges of selected quantitative differences measured between symmetric versus asymmetric CIMR in humans**

	Normal/No MR	Symmetric CIMR	Asymmetric CIMR
Tethering Angles			
α	25°	25°-45°	25°-45°
β	36°	30°-45°	40°-60°
Ratio (β:α)	1.4	1.2-1.9	1.6-3.0
**Tenting**			
Tenting height		9.3 mm	7.7 mm
Tenting area	0.6 cm^2^-0.8 cm^2^	3.0 cm^2^-3.2 cm^2^	4.0 cm^2^-4.1 cm^2^
Tenting volume	2.3 mL	4.4 mL-5.5 mL	2.5 mL-4.3 mL
**PM Anatomy**			
Posterior displacement: ALPM	1.7 cm	3.3 cm	2.5 cm
Posterior displacement: PMPM	1.6 cm	3.1 cm	3.0 cm
Lateral displacement: ALPM	1.2 cm	2.2 cm	2.0 cm
Lateral displacement: PMPM	1.2 cm	2.0 cm	2.3 cm
Apical displacement: ALPM	2.4 cm	3.0 cm	2.9 cm
Apical displacement: PMPM	2.4 cm-2.5 cm	3.1 cm-3.8 cm	3.0 cm
Interpapillary distance	2.1 cm-2.5 cm	3.5 cm-3.8 cm	3.0 cm
**Annulus**			
Height	6-8 cm	8.5 mm	10.3 mm
Area	6.4 cm^2^	11.1 cm^2^-12.3 cm^2^	9.0 cm^2^-10.8 cm^2^
Systolic area change	56%	13.9%	30.7%
**Left Ventricle**			
Ejection fraction	55-70%	37%-41%	44%-44%
EDV (index)	(35-75 mL/m^2^)	210 mL-228 mL (115 mL/m^2^)	184 mL-195 mL (101 mL/m^2^)
ESV (index)	(12-30 mL/m^2^)	135 mL-152 mL (69 mL/m^2^)	102 mL-107 mL (59 mL/m^2^)
Sphericity Index	0.43	0.58-0.66	0.56-0.56
Wall motion score index	1.0	1.6-2.0	1.2-1.8

Data from: Zeng et al. [12, 22]; Gelsomino et al. [23]; Veronesi et al. [19]; Agricola et al. [7].

Wall motion abnormalities are critically important in gauging local LV dysfunction in CIMR: the echocardiographer should identify and quantify wall motion as part of a comprehensive assessment of a global assessment of ischemic burden. Indices of wall motion abnormalities underlying the posteromedial PM insertion are highly important in assessing CIMR. Novel methodologies, including LV basal rotation dynamics as assessed by speckle tracking, further highlight local differences between myocardial function in symmetric and asymmetric phenotypes [10]. Normal systolic rotation may contribute to decreasing the distance from posterior PM head to leaflet and mitral annular contraction. In a multivariable model, impairment of basal rotation was a key predictor of CIMR severity after inferoposterior MI, likely because of less ability of myocardial rotation to reduce adverse tethering lengths and also a contribution to reduction of mitral annular contraction.

#### PM dysfunction

Ischemic and/or systolic PM dysfunction itself does not seem to contribute to CIMR on top of the contribution of PM displacement. Kaul first reported poor overall correlation of reduced PM thickening and MR severity in canines [24]. In a sheep model of CIMR by left circumflex occlusion but with preserved PM blood supply via a perfusion catheter from the aorta, withdrawal of the perfusion catheter caused onset of papillary ischemia as measured by decreased strain rate but was correlated with diminished tethering distances and reduced MR [25]. In humans, there is some evidence that PM dysfunction, as measured by longitudinal systolic strain, actually reduces MR observed after inferior myocardial infarction [26]. Impairment of PM contraction presumably reduces tension on the chordae and paradoxically compensates for the tethering forces exerted by PM misalignment and/or LV dilatation. Novel protocols employing delayed enhancement cardiac magnetic resonance imaging confirmed that while PM infarct was observed in 30% of patients at 4 weeks after first myocardial infarction, neither partial nor complete PM infarct robustly correlated with CIMR [27]. These observations reinforce the notion that geometric PM displacement, and not necessarily systolic function, is the key factor in determining CIMR.

#### Tethering and tenting of the mitral leaflets is the final common pathway mediating leaflet malcoaptation and incomplete closure in CIMR

The aggregate of the abnormal vector forces on the mitral leaflets manifest echocardiographically as incomplete mitral leaflet closure or tenting; as such it represents the common pathway of LV remodelling and PM displacement in CIMR. Various measures of quantifying tethering and tenting are available by routine 2D TTE techniques. The incomplete mitral leaflet closure pattern is often best appreciated in the apical four chamber view, because the mitral annular plane is defined in this view.A single linear measure of “tenting height” – the maximal mid-systolic distance from mitral leaflet tips to the annular plane – reflects the abnormal apical shift of the coaptation zone (Figure 10A). While this measure has been correlated with CIMR severity, tenting height understandably may be different when the tethering forces are directed posterolaterally versus apically for example because height alone it does not account for angle of tethering relative to the annular plane.

The tethering angles define the relationship of the base of the leaflets to the annulus: α represents the angle between annular plane and anterior mitral leaflet and β the angle between annular plane and posterior mitral leaflet [23]. 3D TTE and TEE acquisition of volumetric data sets allows selection of particular imaging slices to calculate tethering angles [22]. Though the exact values depend on methodology and imaging plane selected, higher ratios of posterior angle to anterior angle characterize the asymmetric tenting phenotypes, and also predicts increased MR severity [22].

**Figure 11:**
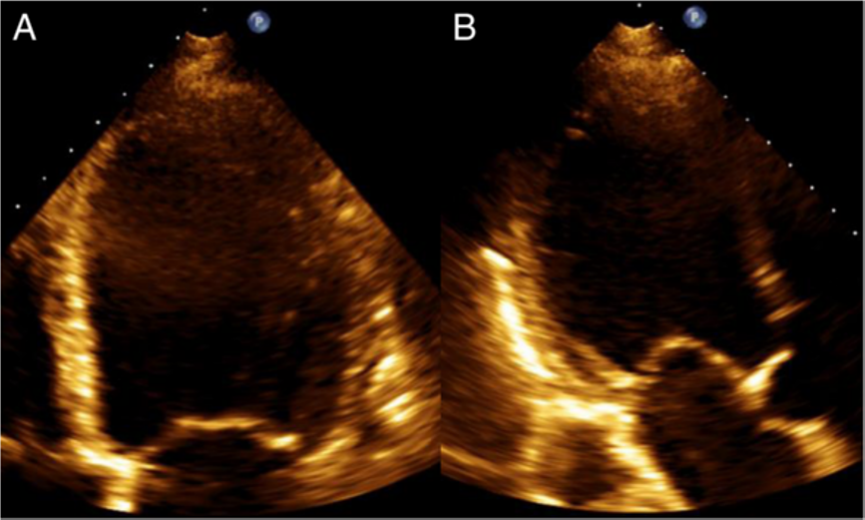
**Symmetric tenting due to ischemic LV dilatation.** These TTE images were obtained from a 72 year-old male with severe multivessel coronary disease and an advanced ischemic cardiomyopathy with LV ejection fraction of 14% and an LV end-diastolic dimension of 71 mm prior to coronary bypass surgery. A phenotype of symmetric tethering is depicted by these mid-systole images obtained from the apical three chamber view **(A)** and apical four chamber view **(B)**. The parasternal long axis view is shown as panel A of Figure 4. Measured in the parasternal long axis image, the tenting height was 1.4 cm, the tenting area was 4.0 cm^2^ and the tethering angles β and α were equal. MR severity was graded as trace. Compared to the patient described in Figure 3, the same tenting height and tenting area were associated with markedly distinct CIMR severity, reiterating that tenting phenotype is of utmost importance in determining severity.

Tenting area provides a more integrative measurement that is less dependent on a particular angle, and also accounts for the geometry of the entire leaflet and not just that at the annular attachment. Tenting area is calculated as the area bounded by the anterior and posterior leaflets and the mitral annular plane (Figure 10); this measurement is performed at mid-systole, when the tenting area would be at a maximum. In the VALIANT-Echo substudy of 341 patients with echocardiographic LV ejection fraction <35% after myocardial infarct, tenting area was the only independent predictor of progressively worsening CIMR based on followup TTE data to a median 24.7 months [28]. Tenting area above a threshold of 4 cm^2^ predicted near 6 fold odds of having moderate or greater MR at the end of followup and an odds ratio of 3.6 for increase in the degree of MR. In patients with LV systolic dysfunction, tenting area was a major determinant of functional MR severity, independent of global LV function, LV volume, and spherical shape. Tenting area itself correlates with linear measures of apical or posterior PM displacements [18]. Extending the analogy of assessment of tenting beyond tenting height and tenting area, tenting volume as defined by 3D echocardiography affords another level of comprehensive measurement of mitral valve deformation. However, the importance of the tenting phenotype must be considered, because even with the same indices of tenting height, area, or volume, an asymmetric CIMR phenotype will likely be associated with more significant MR (Figure 11).

Finally, secondary chordal attachments (basal or strut chordae) to the anterior mitral valve leaflet may exert additional geometric constraints on systolic MV configuration, most commonly manifesting as a bend, or an angle, between the distal and basal portions of the anterior mitral leaflet that further impairs coaptation. This angle can give a qualitative visual clue, assessed as a convexity or concavity in the configuration of the anterior mitral valve leaflet toward the left atrium in the parasternal long axis view in systole, with concavity indicating a bowing into the LV that strongly correlated with CIMR severity [29].

#### Mitral annular dilatation

The mitral annulus has a specialized 3D geometry likened to an ovoid saddle shape that reduces stresses on the leaflets and supports valvular competence [30]. Dilatation of the annulus may occur secondary to either LV or LA dilatation, and while dilatation occurs primarily along the posterior annulus, even the fibrous anterior portion of the mitral annulus may dilate [31, 32]. Additionally, dilatation along the posterior annulus may be asymmetric, with a predilection for the region of the posterior commissure (P_2_ – P_3_ segment).

Annular dilatation can cause an incomplete coaptation pattern due to insufficient available leaflet area. However, the degree of dilatation does not necessarily correlate with the severity of CIMR. Distortion of the native 3D annular geometry to a “flattened” annulus may also contribute to CIMR by changing the leaflet motion. However in a study of lone atrial fibrillation patients with annular dilatation but normal LV chamber size, significant MR was not observed [33]. This is because LV remodelling and dilatation is required to generate tethering forces, though the study did show a weak correlation between functional MR severity and annular area.

Annular dilatation can be measured by anterior and posterior dimensions, annulus area (apical four chamber mitral annulus dimension multiplied by apical two chamber mitral annulus dimension multiplied by π/4) and perhaps with more computationally sophisticated methods such as the MVQ software package (Mitral Valve Quantification, Phillips). Surveillance of mitral annular dilatation is a part of our practice because of a self-propagating cycle of annular dilatation → MR → LV dilatation → annular dilatation. Mitral annular contraction, equal to (diastolic annular area – systolic annular area)/diastolic annular area, has a negative correlation with MR severity in LV systolic dysfunction [18] and in post-infarct MR.

#### Mitral leaflet area

Work by Robert Levine at Massachusetts General Hospital has described 3D echocardiographic methods to compare the areas of the mitral leaflets to the “closing area” and the annular area [34, 35]. In human models of functional MR, the mitral leaflet areas are greater than in patients without dilatation or prior infarct. However, the ratio of the measured mitral leaflet area to the calculated “closing area” is decreased in functional MR. There may a threshold lower ratio which would be consistent with diagnosing a functional MR mechanism; it may be possible in the future to echocardiographically detect, measure and monitor this process as a means of assessing the remodelling response to CIMR. The biologic response that allows the valve to remodel by enlargement and thickening seems to be due to reactivation of embryonic development pathways occurring within the leaflet tissue [36].

### Assessment of CIMR post-therapy

#### Echocardiography post-annuloplasty

The mechanisms responsible for recurrence of CIMR after surgical revascularization and restrictive annuloplasty remain elusive. In some instances, the mechanism is ongoing adverse LV dilatation and spherical remodelling that worsens tethering [37, 38]. In a single center retrospective population of predominantly ischemic MR, preoperative LV end diastolic diameter indexed to body surface area with a cut-off of >3.5 cm/m^2^ predicted recurrence of MR [39]. A greater degree of anterior mitral leaflet tethering angle α, specifically >36.9° (considered the moderate-to-severe or severe quintiles of anterior tethering), regardless of LV dilatation or geometry, conferred a multivariate OR of 3.6 for recurrent MR at 44.7 month follow-up of CIMR patients who underwent surgical revascularization and undersized ring annuloplasty [40]. This is in accord previous results showing α ≥39.5° conferred OR of 3.1 for recurrent MR in a similar population of patients who underwent surgical revascularization and undersized ring annuloplasty [41]. There was also a strong association (OR >4) for lack of LV reverse remodeling post-operatively. The results of this line of analysis underscores that preoperative echocardiography and tethering geometry does predict postoperative outcomes including MR recurrence, LV geometry, and outcome, and thus these should be part of preoperative assessment. Preoperative diastology may also impact postoperative outcome, with transmitral deceleration time <140 ms predictive of MR recurrence, and deceleration time and pulmonary vein systolic:diastolic flow ratio predictive of mortality [42].

Because annuloplasty shifts the coaptation zone more anteriorly, the posteromedial PM location can be further distorted and lie outside the annulus ring; the tethering effect on the posterior leaflet makes it less likely to coapt at the anteriorly shifted coaptation zone [43]. In patients without continued global LV dilatation, recurrent MR is highlighted by adverse anterior leaflet tethering due to bending, as measured by anterior leaflet coaptation area [38].

### Additional imaging techniques

#### Strain imaging

Derangements in peak systolic longitudinal, radial, and circumferential strain measures mirror underlying wall motion abnormalities in both asymmetric or symmetric CIMR [13]. In symmetric CIMR, peak systolic strain was reduced globally, while in asymmetric CIMR phenotypes there was more localized systolic strain derangements in the inferoseptal and inferior territories. While it is not yet clear how strain might add to the diagnosis of CIMR, it could assume a particular role in surgical planning: in a 61 patient CIMR cohort, strain did not improve after surgical revascularization and restrictive annuloplasty in the symmetric group, but did improve at one year in the asymmetric group [13].

#### Exercise echocardiography

Patients with mild (or “progressive”) rest CIMR may exhibit more severe inducible regurgitation as assessed by flow convergence methods [13], and this may represent the etiologies of exertional symptoms [44] and excess mortality seen with CIMR [45]. Exercise physiology exerts multiple effects that bear on the mitral valve apparatus and degree of MR: inotropy increases which augments global and regional LV systolic dysfunction and has the potential to improve mitral valve coaptation geometry; against this, exercise contributes to increased LV systolic pressure and increased chronotropy with shortened systolic time, which contribute to augmented transmitral LV to left atrial pressure gradient [46]. Additionally, exercise-induced ischemia could contribute to new or worsened WMA and tethering, or increased heart rate and altered loading conditions may result in worsening of ventricular mechanics which in setting of underlying akinesis or dyskinesis, result in increased MR. The net change in ischemic MR with exercise depends ultimately on which factor(s) represent the underlying mechanism of the ischemic MR: about one-quarter of CIMR patients show decrease ischemic MR with exercise [47], e.g. those with inferior myocardial infarction who can augment LV function with exercise and who would not have worsened ventricular mechanics.

Exercise may present a method to risk stratify patients with LV systolic dysfunction and mild rest CIMR at rest, as cardiovascular mortality at 19 month followup was predicted by worsening of mild rest CIMR (judged by an increase in EROA ≥13 mm^2^ on a symptom-limited semisupine bicycle exercise test for which beta blockers were held for 24 hours) [47]. In another study of submaximal Bruce protocol treadmill exercise with patients on beta blockers, no rest echocardiographic parameters predicted severity of exercise-induced CIMR by EROA; instead only changes in exercise-induced mitral geometry measured by valve tenting area and coaptation distance represented the independent predictors of ischemic MR severity [48]. Exercise echocardiography may be reasonable in patients with ischemic heart disease and suspected CIMR who report dyspnea disproportionate to rest MR and/or LV dysfunction or who experience pulmonary edema without explained cause, and for whom additional information would answer whether surgery would benefit [13, 46].

#### Transesophageal Echocardiography (TEE)

TEE can be a useful adjunct to TTE for characterizing the mechanism of MR (particularly for intrinsic leaflet pathologies) and mapping anatomic defects. It may help exclude an organic etiology when assessing the patient with CIMR, and also provide better spatial resolution of chordal and leaflet geometric relationships. The use of TEE intraoperatively and post-operatively in the evaluation of MR has been comprehensively reviewed by Sidebotham et al. [49] and Shakil et al. [50]. TEE is important in assessment of patients undergoing surgical revascularization as it provides another opportunity to assess for CIMR. However, because of vasodilating effects of anesthesia, CIMR severity may be underestimated by intraoperative TEE. One proposed tactic to ensure appropriate severity grading is to administer vasopressors to mimic more physiologic afterload conditions. In a single study, the proxy for physiologic afterload was a systolic blood pressure of 160 mmHg though the exact target is debatable; concurrent with vasopressor administration, most patients’ pulmonary artery occlusion pressure rose and only a few patients were administered extra intravenous fluid to combat the venodilating effects of anesthesia [51].

#### Cardiac computed tomography and magnetic resonance

Non-echocardiographic cardiac imaging modalities are being deployed to study CIMR. These techniques may require the patient to remain immobile and flat and to perform breath holds – potential issues for patients with orthopnea due to cardiomyopathy or MR. Computed tomography implies a radiation exposure and magnetic resonance may require significant time as well as specialized equipment. Nevertheless, robust data sets with axial and three dimensional information may be derived which are suitable for a comprehensive classification of the interwoven geometry of the components of the mitral valve apparatus, for example augmented definition of annulus dimensions, annulus height, shape, and tenting height and angles [31, 52, 53] Delayed enhancement cardiac magnetic resonance and CT also offer alternative routes to more precise definition of region of PM and LV myocardial infarct [27], and are thus useful to establish the underlying ischemic etiology of MR and also define myocardial viability which may impact treatment decision-making [2]. The role of computed tomography and magnetic resonance remains to be defined.

## Conclusion

CIMR is the post-infarct pathophysiologic result of incomplete mitral valve coaptation due to global or local geometric deformity of the LV that generates apical displacement of normal mitral valve leaflets. Echocardiographic techniques can quantitate CIMR primarily by indices of leaflet tethering and tenting, effectively integrating the effects of multiple disparate forces. Because CIMR may beget CIMR through mechanisms of increased volume loading on the LV and annular dilatation, precise echocardiographic diagnosis and followup are essential. Improved echocardiographic assessments will augment our understanding of the etiologies CIMR, and translate to appropriate, mechanistically-targeted therapy. The typical therapy of CIMR has been restrictive mitral annuloplasty, but reshaping the annulus cannot alone solve the problem when the ultimate etiologic lesion is in ventricular geometry. This dilemma is appreciated in practice as it is not proven that current treatments of CIMR either boosts survival, helps LV reverse remodeling, or improves symptoms. Better definitions and improved phenotyping of CIMR will permit robust randomized controlled trials of surgical therapies [54], as well as refinements in percutaneous device therapies such as CRT (which reduces tethering and modifies timing of LV systolic forces) [55] and injectable polymers to reshape the posteromedial PM [56], novel surgical approaches (e.g. targeted surgical approaches such as LVplasty, annuloplasty and chordal cutting depending on the patient’s specific CIMR mechanism), and experimental devices to improve PM geometry [57].

## Authors’ information

JH is an associate professor of medicine at Harvard Medical School and the associate director of the echocardiography laboratory at Massachusetts General Hospital. She has participated on several writing group committees for American Society of Echocardiography guidelines, including those on valvular disease.

## Electronic supplementary material

Additional file 1: **TTE image of asymmetric CIMR developing after inferoposterior myocardial infarction.** This video shows a TTE parasternal long axis loop taken from the same patient as described in Figure 3, and it demonstrates the inferobasal aneurysm and asymmetric tenting due to more severe tethering of the posterior mitral leaflet than the anterior mitral leaflet (the underlying rhythm is atrial fibrillation and the effects of variable heart rate must be appreciated by the echocardiographer as it may confound analysis). (ZIP 5 MB)

## Abbreviations

2D: Two-dimensional
3D: Three-dimensional
CIMR: Chronic ischemic mitral regurgitation
EROA: Effective regurgitant orifice area
GDMT: Guideline directed medical therapy
LA: Left atrium
LV: Left ventricle
mL: Milliliters
MR: Mitral regurgitation
MV: Mitral valve
OR: Odds ratio
PISA: Proximal isovelocity surface area
PM: Papillary muscle
RVol: Regurgitant volume
TEE: Transesophageal echocardiography
TTE: Transthoracic echocardiography
TVI: Time velocity integral
VC: Vena contracta
WMA: Wall motion abnormalities.
